# Long-term Pegvisomant Therapy of Acromegaly: Effects on Bone Density, Turnover and Microstructure Using HRpQCT

**DOI:** 10.1210/jendso/bvae079

**Published:** 2024-04-17

**Authors:** Adriana P Kuker, Sanchita Agarwal, Elizabeth Shane, Juliana Bicca, Eliza B Geer, Serge Cremers, Elzbieta Dworakowski, Adi Cohen, Thomas L Nickolas, Emily M Stein, Pamela U Freda

**Affiliations:** Department of Medicine, Vagelos College of Physicians & Surgeons, Columbia University, New York, NY 10032, USA; Department of Medicine, Vagelos College of Physicians & Surgeons, Columbia University, New York, NY 10032, USA; Department of Medicine, Vagelos College of Physicians & Surgeons, Columbia University, New York, NY 10032, USA; Plenicca Clinic, Sao Paolo 05688-021, Brazil; Department of Medicine, Memorial Sloan Kettering Cancer Center, New York, NY 10065, USA; Department of Neurosurgery, Memorial Sloan Kettering Cancer Center, New York, NY, 10065, USA; Department of Medicine, Vagelos College of Physicians & Surgeons, Columbia University, New York, NY 10032, USA; Department of Pathology and Cell Biology, Vagelos College of Physicians & Surgeons, Columbia University, New York, NY 10032, USA; Department of Medicine, Vagelos College of Physicians & Surgeons, Columbia University, New York, NY 10032, USA; Department of Medicine, Vagelos College of Physicians & Surgeons, Columbia University, New York, NY 10032, USA; Department of Medicine, Vagelos College of Physicians & Surgeons, Columbia University, New York, NY 10032, USA; Endocrinology and Metabolic Bone Diseases, Hospital for Special Surgery, New York, NY 10021, USA; Department of Medicine, Vagelos College of Physicians & Surgeons, Columbia University, New York, NY 10032, USA

**Keywords:** acromegaly, HRpQCT, bone

## Abstract

**Context:**

Fracture rate is increased in patients with active acromegaly and those in remission. Abnormalities of bone microstructure are present in patients with active disease and persist despite biochemical control after surgery. Effects of treatment with the GH receptor antagonist pegvisomant on bone microstructure were unknown.

**Methods:**

We studied 25 patients with acromegaly (15 men, 10 women). In 20, we evaluated areal bone mineral density (BMD) by dual-energy X-ray absorptiometry and bone turnover markers (BTMs) longitudinally, before and during pegvisomant treatment. After long-term pegvisomant in 17, we cross-sectionally assessed volumetric BMD, microarchitecture, stiffness, and failure load of the distal radius and tibia using high-resolution peripheral quantitative computed tomography (HRpQCT) and compared these results to those of healthy controls and 2 comparison groups of nonpegvisomant-treated acromegaly patients, remission, and active disease, matched for other therapies and characteristics.

**Results:**

In the longitudinal study, areal BMD improved at the lumbar spine but decreased at the hip in men after a median ∼7 years of pegvisomant. In the cross-sectional study, patients on a median ∼9 years of pegvisomant had significantly larger bones, lower trabecular and cortical volumetric density, and disrupted trabecular microarchitecture compared to healthy controls. Microstructure was similar in the pegvisomant and acromegaly comparison groups. BTMs were lowered, then stable over time.

**Conclusion:**

In this, the first study to examine bone microstructure in pegvisomant-treated acromegaly, we found deficits in volumetric BMD and microarchitecture of the peripheral skeleton. BTM levels remained stable with long-term therapy. Deficits in bone quality identified by HRpQCT may play a role in the pathogenesis of fragility in treated acromegaly.

GH and IGF-1 are important regulators of bone metabolism and remodeling. When in excess in acromegaly, however, they lead to high bone turnover, and this, along with hypogonadism and other factors, is believed to cause the poor bone quality observed in these patients [1]. Poor bone quality is evidenced by the finding of an increased rate of vertebral fracture (VF) in patients with active acromegaly that persists despite long-term biochemical control [2, 3]. Unfortunately, areal bone mineral density (aBMD) measured by dual-energy X-ray absorptiometry (DXA) is an unreliable indicator of this fragility [4, 5]. In other populations, high-resolution peripheral quantitative tomography (HRpQCT) directly identifies changes in bone microstructure related to fragility independently of aBMD [6-9]. In acromegaly patients, HRpQCT has revealed deficits in bone microstructure that remain in patients in remission after surgery compared to nonacromegaly controls [10]. Although treatment of acromegaly with the GH receptor (GHR) antagonist pegvisomant achieves a high rate of long-term biochemical control and can improve acromegaly comorbidities [11-14], little is known about its effects on the bone disease of acromegaly. While short term studies have shown that pegvisomant therapy lowers the high serum levels of bone turnover markers (BTMs) that are characteristic of active acromegaly [15, 16] and aBMD may improve [17], VF rate appears to remain high in pegvisomant-treated patients [18]. Since it is unclear how well circulating IGF-1 levels reflect tissue-level GH/IGF-1 exposure, it is of clinical interest to ensure that long-term GHR antagonism has effects on bone that are comparable to other acromegaly therapies and does not oversuppress bone turnover or reduce aBMD, such as might occur with GH deficiency [19]. The effects of pegvisomant on bone microstructure have not been studied, and its long-term effects on bone turnover and density remain unknown. Our primary objective, therefore, was to determine the consequences of long-term acromegaly treatment with pegvisomant on bone density and quality. To do this, we first studied, longitudinally, changes in aBMD in 20 patients starting pegvisomant therapy. We then, in a cross-sectional study, assessed true volumetric BMD, microarchitecture, and stiffness of the distal radius and tibia using HRpQCT in 17 patients on long-term pegvisomant therapy compared to nonacromegaly healthy controls and to 2 matched groups of acromegaly patients not receiving pegvisomant. The prevalence of VFs was examined in this cross-sectional study. We also examined the effects of pegvisomant therapy on BTMs, both changes in the longitudinal study and levels during long-term therapy in the cross-sectional study.

## Methods

### Subjects

#### Acromegaly patients

##### Longitudinal study group

We longitudinally studied 20 acromegaly patients (12 men, 8 women), aged 48.1 ± 10.5 years (range 36–77 years) who were beginning treatment with pegvisomant by DXA and BTM testing. Characteristics of this study group are shown in Table 1. Prior to starting pegvisomant, all had noncurative transsphenoidal surgery, 5 also had radiotherapy and 19 had received other medical therapies that did not normalize their IGF-1 levels. Six patients had diabetes mellitus: 5 were treated with oral agents and 1 with insulin. Of the 20 patients, 17 patients were treated with pegvisomant monotherapy after switching from somatostatin-receptor ligands (SRLs) and 3 added pegvisomant to stable doses of SRLs that were continued for the duration of the follow-up period (Table 1). Twelve patients participated in longitudinal BTM testing. Testing was performed as part of a longitudinal, single-center study that performed DXA, BTMs, and clinical and endocrine surveillance of patients beginning pegvisomant therapy for acromegaly from 2003 to 2018. Results of DXA body composition testing, other than bone, were reported previously for 18 patients [20].

**Table 1.** Longitudinal DXA and BTM study: clinical, endocrine, and DXA data before and on pegvisomant therapy in 20 patients

	Women (n = 8)			Men (n = 12)			All (n = 20)		
	Baseline	Last follow-up	*P*-value	Baseline	Last follow-up	*P*-value	Baseline	Last follow-up	*P*-value
Age at DXA (yr) (range)	48.4 ± 8 (36–58)	54.4 ± 9.8 (41–69)	n/a	47.9 ± 12.2 (37–77)	55.8 ± 10.7 (40–79)	n/a	48.1 ± 10.5 (36–77)	55.3 ± 10 (40–70)	n/a
Height (cm)	164 ± 3.6	164 ± 3.7	.5	180 ± 6.8	180 ± 7	.36	174 ± 9.8	173.6 ± 9.8	.68
Weight (kg)	75.8 ± 13	76 ± 12	.87	107.5 ± 17	106.9 ± 16	.93	94.8 ± 22	94.5 ± 21	.97
BMI (kg/m^2^)	28 ± 4.7	28 ± 4.5	.89	33 ± 4.4	33 ± 4.3	.93	31 ± 5	31 ± 4.9	.97
Prior acromegaly therapies (no.)	S(8), LAR(6), CAB (4), SOM (1), RT (2)	—	n/a	S (12), LAR (9), CAB (5), BC (2), SOM (1), PAS (2), RT (3)	—	n/a	S (20), LAR (15), CAB (9), BC (2), SOM (2), PAS (2), RT (5)	—	n/a
Acromegaly cotherapy	—	—	n/a	LAR (2), OCT (1)	LAR (2), OCT (1)	>.99	LAR (2), OCT (1)	LAR (2), OCT (1)	>.99
Duration of PEGV therapy, baseline to follow-up (yr) (median, range)	—	5.1 (1.7–11.7)	n/a	—	7.9 (1.8–15.9)	n/a	—	7.3 (1.7–15.9)	n/a
Gonadal function (no.)	E (4), PM (3), H (1)	E (2), PM (5), H (1)	ND	E (7), H (1), HR (4)	E (7), H (1), HR (4)	ND	E (11), PM (3), H (2), HR (4)	E (9), PM (5), H (2), HR (4)	ND
Hormone replacements*^a^*	0	0	>.99	T4 (2), HC (1)	T4 (2), HC (1)	>.99	T4 (2), HC (1)	T4 (2), HC (1)	>.99
Diabetes	2	2	>.99	4	4	>.99	6	6	>.99
Smoking	0	0	>.99	2	2	>.99	2	2	>.99
IGF-1 (µg/L)	406 ± 196	216 ± 96	.**009**	555 ± 156	198 ± 72	**<**.**001**	495 ± 184	206 ± 80	**<**.**001**
IGF-1% ULN	162 ± 79	87 ± 25	.**02**	218 ± 59	85 ± 27	**<**.**001**	196 ± 72	86 ± 26	**<**.**001**
IGF-1 < ULN (%)/ < 1.3X ULN (%)	0	87.5/100	n/a	0	91/91	n/a	0	90/95	n/a
Vitamin D status (%)	Borderline (37.5), normal (62.5)	Borderline (37.5), normal (62.5)	>.99*	Borderline (42), normal (58)	Borderline (42), normal (58)	>.99	Borderline (40), normal (60)	Borderline (40), normal (60)	>.99
DXA									
Total body									
Z-score	0.9 ± 1.4	1 ± 1.5	.49	−0.2 ± 0.9	0.3 ± 0.9	.06	0.2 ± 1.2	0.5 ± 1.2	.**04**
T-score	0.9 ± 1.3	0.9 ± 1.9	.90	0.6 ± 0.9	0.9 ± 1.2	.15	0.8 ± 1	0.9 4 ± 1.5	.35
aBMD(g/cm^2^)	1.22 ± 0.11	1.20 ± 0.15	.88	1.27 ± 0.07	1.29 ± 0.09	.15	1.25 ± 0.09	1.26 ± 0.13	.33
L1-L4									
Z-score	0.2 ± 0.8	0.2 ± 0.7	.81	−0.4 ± 1.2	0.5 ± 1.5	.**01**	−0.2 ± 1.1	0.4 ± 1.2	.**04**
T-score	−0.2 ± 1.5	−0.3 ± 1.4	.90	0.1 ± 1.6	0.8 ± 1.6	.06	−0.1 ± 1.6	0.2 ± 1.6	.21
aBMD(g/cm^2^)	1.16 ± 0.22	1.17 ± 0.19	.88	1.25 ± 0.20	1.34 ± 0.21	.04	1.19 ± 0.21	1.25 ± 0.21	.14
Total hip									
Z-score	0.7 ± 1.3	0.6 ± 1.4	.68	0.3 ± 0.9	0.07 ± 0.8	.10	0.5 ± 1	0.3 ± 1	.13
T-score	0.4 ± 1.2	−0.04 ± 1.7	.34	0.4 ± 1.1	0 ± 0.9	.**04**	0.4 ± 1.1	−0.01 ± 1.1	.**03**
aBMD(g/cm^2^)	1.06 ± 0.15	1 ± 0.21	.31	1.15 ± 0.17	1.09 ± 0.11	.94	1.1 ± 0.16	1.06 ± 0.15	.**035**
Femoral neck									
Z-score	0.5 ± 0.6	0.1 ± 0.4	.31	1.1 ± 0.9	0.7 ± 0.9	.29	0.8 ± 0.9	0.5 ± 0.8	.13
T-score	−0.2 ± 0.7	−0.8 ± 0.9	.21	1.2 ± 1.1	0.3 ± 1.1	.**01**	0.7 ± 1.2	−0.1 ± 1.1	.**003**
aBMD(g/cm^2^)	0.98 ± 0.15	0.93 ± 0.12	.49	1.15 ± 0.2	1.1 ± 0.1	.**01**	1.1 ± 0.16	1.0 ± 0.14	.**01**
One-third radius									
Z-score	0.1 ± 1.1	−0.3 ± 1.3	.28	−0.2 ± 0.9	0.4 ± 1.1	.**02**	−0.1 ± 0.9	0.1 ± 1.2	.31
T-score	−0.5 ± 1.2	−1.2 ± 1.7	.19	−0.6 ± 1.3	−0.5 ± 1.7	.65	−0.6 ± 1.3	−0.7 ± 1.7	.71
aBMD(g/cm^2^)	0.35 ± 0.76	0.3 ± 0.74	.19	0.43 ± 1.03	0.76 ± 0.14	.30	0.4 ± 0.9	0.61 ± 0.47	.33
UD radius									
Z-score	1.1 ± 3.2	1.5 ± 3.9	.59	1.8 ± 2.3	2.5 ± 2.3	.06	1.6 ± 2.5	2.2 ± 2.8	.06
T-score	0.7 ± 3.6	0.7 ± 4.7	.9	1.7 ± 2.6	2.2 ± 2.6	.16	1.4 ± 2.8	1.7 ± 3.3	.35
aBMD(g/cm^2^)	0.42 ± 0.13	0.4 ± 0.17	.73	0.49 ± 0.11	0.53 ± 0.12	.**004**	0.47 ± 0.11	0.49 ± 0.14	.26
Osteoporosis (%)	12.5	12.5	>.99	8.3	8.3	>.99	12	12	>.99
Osteopenia (%)	37.5	50	>.99	41.7	33.3	>.99	47	47	>.99

Data are shown for women and men separately and for all patients combined. Data are mean ± SD unless otherwise indicated. Significant *P*-values are shown in bold text.

Abbreviations: aBMD, areal bone mineral density; BC, bromocriptine; BMI, body mass index; CAB, cabergoline; DXA, dual-energy X-ray absorptiometry; E, eugonadal; GC, oral glucocorticoid; H, hypogonadal (premenopausal women or men) without replacement therapy; HR, hypogonadal on replacement therapy; LAR, octreotide LAR; n/a, not applicable; ND, not determined; ns, nonsignificant; OCT, short-acting octreotide; PAS, pasireotide LAR; PEGV, pegvisomant; PM, postmenopausal on no replacement therapy; RT, radiotherapy; S, surgery; SOM, somatuline depot; T4, levothyroxine; UD, ultradistal; yr, years.

^*a*^Number of subjects.

##### Cross-sectional study group

**Figure 1.**
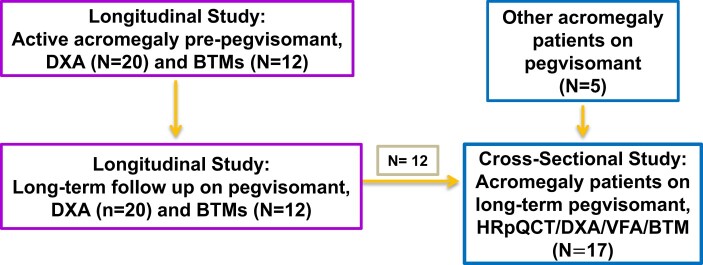
Study design overview: in the prospective, longitudinal study (left), patients with active acromegaly who were beginning pegvisomant therapy were studied serially by DXA (n = 20) and levels of BTMs (n = 12). In a cross-sectional study (right), 17 patients on long-term pegvisomant therapy, 12 who were also in the longitudinal study and 5 others on pegvisomant therapy, were studied by HRpQCT, DXA/VFA, and BTMs. Abbreviations: BTM, bone turnover marker; DXA, dual-energy X-ray absorptiometry; high-resolution peripheral quantitative computed tomography; VFA, vertebral fracture analysis.

We studied 17 patients with acromegaly on long-term pegvisomant therapy cross-sectionally by HRpQCT, DXA/vertebral fracture analysis (VFA), and BTM measurements. Characteristics of this group are shown in Table 2. It included 12 patients who had participated in the longitudinal DXA study and 5 additional patients on long-term pegvisomant therapy (Fig. 1). Prior to starting pegvisomant, all had noncurative transsphenoidal surgery, 5 also had radiotherapy, and 16 had received other medical therapies that did not normalize their IGF-1 levels. Six patients had diabetes mellitus: 5 were treated with oral agents and 1 with insulin. Thirteen had switched from other medical therapies to pegvisomant monotherapy, and 4 were on a combination of pegvisomant and SRL therapy (Table 2). The cross-sectional study group was recruited from among patients with acromegaly seen at our centers from 2016 to 2019 who had been receiving pegvisomant for at least 1 year. Of those eligible, 1 who had a predominantly prolactin-secreting lactotroph/somatotroph tumor and 1 who had a history of osteoporosis pharmacotherapy were excluded and 13 declined participation.

**Table 2.** Cross-sectional study: clinical and endocrine characteristics in patients with acromegaly on long-term PEGV therapy studied cross-sectionally and in the matched comparison groups of acromegaly in remission or active disease

	PEGV-treated group (n = 17)	Acromegaly remission comparison group (n = 17)	Active acromegaly comparison group (n = 17)	*P*-value PEGV vs remission	*P*-value PEGV vs active
Age (yr) (range)	55.7 ± 12.6 (26–79)	56.6 ± 12.3 (26.8–71.8)	53 ± 11 (27–73)	.83	.56
Sex (men/women)	11/6	11/6	11/6	n/a	n/a
BMI (kg/m^2^)	32 ± 5	31.5 ± 4.7	31.4 ± 7.3	.88	.76
Duration of PEGV treatment, yr. (median, range)	11 (3–15)	—	—	n/a	n/a
Weekly PEGV dose, mg (range)	165 ± 126 (25–255)	—	—	n/a	n/a
Years of PEGV vs years from surgery	9.88 ± 4.14	9.3 ± 6.1	9.84 ± 7.13	.76	.98
Prior surgery*^a^*	17	17	10	>.99	.007
Radiotherapy*^a^*	5	2	1	.39	.17
Newly diagnosed*^a^*	—	—	7	n/a	n/a
Years from surgery	17.3 ± 8.2	9.33 ± 6.16	9.84 ± 7.13	.**003**	.**02**
Concurrent acromegaly therapy*^a^*	LAR (1), LAR/CAB (1), SOM/CAB (1), PAS (1)	LAR (3), LAR/CAB (1)	LAR (1), LAR/CAB (2), SOM (1)	>.99	>.99
Prior acromegaly medical therapy	LAR (14), SOM (2), CAB (5), PAS (1)	—	LAR (2), LAR/BC (1), BC (1)	**<**.**0001**	**<**.**0001**
Gonadal function and replacements	E (6), PM (5), H (1), HR (5)	E (8), PM (4), H (1), HR (4)	E (7), PM (4), H (2), HR (3)	>.99*^b^*	>.99*^b^*
Glucocorticoid replacement*^a^*	2	1	1	>.99	>.99
Thyroxine replacements*^a^*	4	4	2	>.99	.66
Hypertension*^a^*	11	10	9	>.99	.73
Sleep apnea*^a^*	5	6	8	>.99	.48
Diabetes*^a^*	6	1	3	.09	.44
Lipid-lowering therapy*^a^*	9	6	8	.49	>.99
Joint replacement*^a^*	3	3	3	>.99	>.99
Smoking*^a^*	3 former	6 active, 3 former	2 active, 1 former	.07	>.99
IGF-1 (µg/L)	182 ± 38	187 ± 57	563 ± 294	.18	**<**.**0001**
IGF-1% ULN (%)	85 ± 15	81 ± 20	232 ± 115	.43	**<**.**0001**
IGF-1 < ULN (%)/ < 1 .3X ULN (%)	88/100	78/100	0	.66	n/a
Average IGF-1% ULN per year from diagnosis	112 ± 43	90 ± 58	190 ± 90	.15	.**002**
N-mid OCN (ng/mL)	15 ± 10	18.4 ± 16.8	39.2 ± 28.4	.16	.**001**
CTX (ng/mL)	0.52 ± 0.39	0.65 ± 0.57	1.07 ± 0.51	.43	.**0005**
Vitamin D status (%)	Borderline (47), normal (53)	Borderline (41), normal (59)	Low (17), borderline (42), normal (41)	>.99	>.99
Baecke Physical Activity Score, median (range)	12 (8.25–24.1)	12.8 (6.75–31.2)	12.7 (7.5–29.4)	.77	.67
Osteopenia (%)	**41**	64	53	.30	.73
Osteoporosis (%)	**12**	6	23	.99	.66

Data are mean ± SD unless otherwise indicated. Vitamin D status, low <20 ng/mL, borderline 20-30 ng/nL, normal >30 ng/mL. Significant *P*-values are shown in bold text.

Abbreviations: aBMD, areal bone mineral density; BC, bromocriptine; BMI, body mass index; CAB, cabergoline; CTX, C-terminal telopeptides of type 1 collagen; E, eugonadal; H, hypogonadal (premenopausal women or men) without replacement therapy; HR, hypogonadal on replacement therapy; LAR, octreotide LAR; n/a, not applicable; ND, not determined; ns, nonsignificant; OCN, osteocalcin; PAS, pasireotide LAR; PEGV, pegvisomant; PM, postmenopausal on no replacement therapy; S, surgery; SOM, somatuline depot; ULN, upper limit of normal; yr, year.

^*a*^Number of subjects.

^*b*^Comparison of proportions hypogonadal between groups.

Results of the skeletal assessments and BTM measurements in pegvisomant-treated patients have not been reported previously.

#### Acromegaly comparison groups

Cross-sectional HRpQCT data were compared to that same testing performed in 2 groups of matched acromegaly patients who were not treated with pegvisomant, 1 group of patients in remission and another with active disease (Table 2). Data for these patients were reported in our prior study [10]. The groups were matched to the pegvisomant-treated patients for sex, age, body mass index (BMI), and years of pegvisomant therapy or since surgery and as close as possible for concomitant acromegaly medical therapy at the time of HRpQCT testing. The remission group was also matched for IGF-1% upper limit of normal (ULN) at the time of HRpQCT testing. Groups were similar with regard to gonadal and other pituitary function. Table 2 shows the detailed characteristics of these 2 comparison groups and their comparability to the pegvisomant-treated study group. The pegvisomant-treated group did have evidence of a longer duration of acromegaly in that more time had elapsed since diagnosis for them than for the comparison groups.

For all acromegaly patients, the disease had been diagnosed based on an IGF-1 level above the age-adjusted normal range, clinical characteristics of the disease, and eventual pathological confirmation of a somatotroph pituitary tumor. Patients with hypopituitarism were on stable doses of replacement therapies for ≥3 months prior to testing, with the exception that some with hypogonadism were not replaced. None had an active malignancy, renal or liver disease, hyper- or hypocalcemia, osteoporosis pharmacotherapy (prior or during the study period), recent pregnancy or lactation, or use of supraphysiologic glucocorticoids at baseline or during the period of follow-up. They were not prescribed any particular calcium or vitamin D supplements nor was physical activity regulated.

#### Nonacromegaly healthy controls

HRpQCT results were also compared to those of the same testing performed in healthy controls: 41 women and men who were from 3 cohorts previously studied by Shane, Cohen, Nickolas, and Stein [21-24] and 11 healthy men who were included in our recent study [10]. Healthy controls did not have diabetes mellitus. Each acromegaly patient was matched to 2 to 3 controls for sex, age ± 5 years, and BMI ± 5 kg/m^2^. Controls had a mean BMI of 29.9 ± 2.8 kg/m^2^ (*P* = .19 vs acromegaly) and an age of 54.8 ± 9.7 (*P* = .81 vs acromegaly).

All subjects were ambulatory outpatients with normal renal function and no liver disease. The study was approved by the Institutional Review Board of Columbia University Medical Center, and all subjects gave written informed consent before participation.

### Study Design

An overview of the study design is shown in Fig. 1. For the longitudinal study, all 20 patients participated in 2 to 5 visits that took place from before to up to 14 years after starting pegvisomant. At each visit, patients underwent anthropometric measurements (body weight by a digital scale to the nearest 0.01 kg and height by a stadiometer to the nearest 0.5 cm), a DXA test, and completion of a medical history questionnaire. Six of these patients had fasting (morning) blood sampling for BTMs and IGF-1 levels performed before and at 4, 8, 12, 24, 36, and 48 weeks after starting pegvisomant, and 6 had these performed before and after 1 (n = 3), 2 (n = 4), 4 (n = 3), 5 (n = 1), 8 (n = 1), and 11 (n = 3) years of pegvisomant therapy.

For the cross-sectional study, patients participated in 1 visit at which they underwent anthropometrics, HRpQCT, DXA (with VFA), BTM testing, completed history, and Baecke physical activity [25] questionnaires and had fasting(morning) blood sampling. All blood samples were frozen at −80°C in multiple aliquots and later assayed for measurements of IGF-1 and markers of bone and mineral metabolism.

####  

##### Hormone and bone marker assays

IGF-1 was measured from 2003 to 2005 by radioimmunoassay (Nichols Institute Diagnostics, San Juan Capistrano, CA) calibrated to the World Health Organization’s First International Reference Reagent 1988, IGF-I 87/518, from 2005-2016 by chemiluminescent immunoassay IMMULITE (Siemens), calibrated to World Health Organization IRR NIBSC code 87/518 and from 2016 to 2020 by a chemiluminescent immunoassay (Immunodiagnostic Systems-iSYS) (RRID:AB_2756880) calibrated to recombinant standard 02/254, as described previously [20]. Mineral metabolism and BTMs markers were measured on morning, fasting blood samples.

For the longitudinal 48-week bone marker analysis, bone-specific alkaline phosphatase (RRID:AB_3095588), osteocalcin (OCN) (RRID:AB_2756880), and osteoprotegerin (OPG) (RRID:AB_3096047) levels were measured by ELISAs (Nordic Bioscience, Herlev, Denmark); intact N-terminal propeptide of type I procollagen was measured by competitive radioimmunoassay (Orion Diagnostics Oy, Espoo, Finland); serum cross-linked N-telopeptides of type 1 collagen was measured by ELISA (Wampole Laboratories, Princeton, NJ) (RRID:AB_3096049); intact PTH was measured by immunoradiometric assay (Scantibodies Laboratory, Inc., Santee, CA); and tartrate-resistant acid phosphatase 5b was measured by ELISA (Immunodiagnostic Systems, Tyne and Wear, UK) (RRID:AB_3096044). Serum calcium and phosphate were measured by standard auto-analyzer techniques. For the long-term longitudinal and cross-sectional BTM analyses, C-terminal telopeptides of type 1 collagen (CTX) (Serum CrossLaps CTX-I) (RRID:AB_3075342) and N-mid OCN (RRID:AB_3095994) were measured by ELISAs (Immunodiagnostic Systems).

## Imaging Methods

### Dual-energy X-ray absorptiometry

aBMD of the total body (n = 20), lumbar spine (LS) (n = 15), total hip (TH) (n = 15), femoral neck (FN) (n = 15), one-third radius (1/3R) (n = 15), and ultradistal radius (UDR) (n = 12) of the nondominant forearm were measured by DXA (software version 11.4; GE Lunar Prodigy Advance, Madison WI) in the Metabolic Bone Disease Unit. The same densitometer with the same software and scan speed was used for all visits and scanning was conducted by International Society for Clinical Densitometry-certified and trained technicians who matched identical regions of interest for baseline and follow-ups. Coefficient of variation is 0.68% for the spine, 1.36% for the TH, and 0.7% for the radius.

VFA was performed on the 17 patients in the cross-sectional HRpQCT study group (Table 2). Lateral images of the thoracolumbar spine were acquired at time of DXA with the patient in the left lateral decubitus position. Each image was inspected to rule out nonosteoporotic deformities. VFs were diagnosed on visual inspection using the semiquantitative technique of Genant et al [26] and graded as grade 1 (mild): 20% to 25% reduction in height and 10% to 20% reduction in vertebral body area; grade 2 (moderate): >25% to 40% reduction in height with ≥ 20% to 40% reduction in the area of the vertebral body area; and grade 3 (severe): ≥ 40% reduction in height and vertebral body area [26].

HRpQCT was performed using the first-generation (XCT1; 82 µm; n = 4 scans) and subsequently the second-generation scanner (61 µm; n = 19 scans; Scanco Medical AG, Switzerland). Scans are acquired at the nondominant distal radius and tibia unless there is contraindication. Briefly, the region of interest is defined on a scout film by placing the reference line at the distal endplate of the radius or tibia and a series of parallel slices are acquired at a fixed offset. Attenuation data are converted to equivalent hydroxyapatite densities. The manufacturer's phantom is scanned regularly for quality control. Scans are scored for motion on a scale of 1 (no motion) to 5 (significant blurring of the periosteal surface, discontinuities in the cortical shell, or streaking in the soft tissue). Images with a motion score of 4 to 5 are excluded from analyses. The standard HRpQCT analysis methods we use have been described, validated, and applied in publications [24, 27-30]. We use the manufacturer's standard method to filter and binarize the HRpQCT images [31]. To segment the cortical and trabecular regions, we use an automatic segmentation algorithm [32]. Due to differences in scanner generations, all data from the second-generation device were calibrated to XCT1 using equations from an extensive cross-calibration study at our center [33]. Our in vivo short-term reproducibility (root mean square-coefficient of variation) for XCT1 measures is <1.06% for all density and <5.2% for all structural parameters [30]. Bone strength was also estimated from HRpQCT images by finite element analysis based on the voxel conversion approach [34, 35]. We simulated uniaxial compression on each radius and tibia model up to 1% strain using a homogeneous Young's modulus of 6829 Mpa and Poisson's ratio of 0.3 [36]. We use a custom finite element analysis solver (Version 6.0; Numerics88, Calgary, Alberta) on a desktop workstation (Linux Ubuntu 12.10, 2 × 6-core Intel Xenon, 64GB RAM) to solve the models [37, 38]. We estimated bone stiffness (Stiffness, N/mm), a surrogate for bone strength, and failure load (F.load, N). Cortical porosity, Stiffness, and F.load were measured only in acromegaly patients.

## Statistical Analysis

Continuous variables were summarized by mean ± SD for normally distributed and as median and range for nonnormally distributed variables. For the longitudinal study analyses, baseline to follow-up aBMD and BTM data were compared by paired *t*-test, and clinical and endocrine data were compared by paired *t*-test or Wilcoxon signed-rank test. For the cross-sectional analyses, HRpQCT parameters, aBMD, and BTMs were compared in acromegaly patients to matched controls and to the remission and active acromegaly comparisons group by independent *t*-test, and clinical and endocrine data were compared by independent *t*-test or Mann–Whitney test as appropriate. Using these methods, post hoc analyses were conducted to compare pegvisomant-treated to controls and acromegaly comparison groups in men and women separately, to compare pegvisomant treated to controls after exclusion of patients with diabetes and longitudinal DXA changes in the 14 patients on pegvisomant monotherapy. Using the cross-sectional data, multiple linear regression models were constructed to examine duration of pegvisomant therapy or %ULN IGF-1 as predictors of HRpQCT parameters after adjustment for sex, age, gonadal function, and BMI and to examine all these as predictors of VF. *P*-values < .05 were considered significant. Statistical analyses were performed using GraphPad Prism version 9 for Mac.

## Results

### Longitudinal Study

#### DXA

aBMD was assessed longitudinally over ∼7 years (median) of pegvisomant treatment (Table 1). Ten patients had 2 scans over 2.3(1.7-14) (median, range) years, 6 patients had 3 to 4 scans over 6.4 (1.9-11) years, and 4 patients had 5 scans over 9.9 (7.8-13) years of pegvisomant therapy. The distribution of length of DXA follow-up was similar in men and women. From initial to last follow-up DXA, men had increases in LS Z-score (*P* = .01) and aBMD (*P* = .04), 1/3R Z-score (*P* = .02), and UDR aBMD (*P* = .004) and decreases in TH T-score (*P* = .02), FN T-score (*P* = .007), and aBMD (*P* = .007). In the women, there were only trends for decreases in TH and FN T-scores, but no significant changes occurred at any sites. Inspection of patterns of change in the 10 patients who had 3 to 5 scans over time showed gradual, consistent patterns of change and did not suggest increases then decreases or vice versa. All changes were similar after exclusion of the 3 patients on pegvisomant-SRL combination therapy. At baseline, osteoporosis was present at 1 or more sites in 3 patients (2 men, 1 woman) and osteopenia in 8 patients (5 men, 3 women). At follow-up, 3 patients had osteoporosis (2 men, 1 woman) and 8 had osteopenia (4 men, 4 women). One postmenopausal woman transitioned from osteopenia to osteoporosis at the 1/3R over 7 years on pegvisomant; she also had UDR osteoporosis at baseline and follow-up.

#### Bone markers

In the patients who had bone markers measured ∼monthly over the first 48 weeks of pegvisomant treatment, formation markers, bone-specific alkaline phosphatase, OCN, and intact N-terminal propeptide of type I procollagen decreased significantly (Table 3). Of the resorption markers, serum cross-linked N-telopeptides of type 1 collagen showed only a trend to fall, and tartrate-resistant acid phosphatase 5b did not change. PTH showed a trend to increase early, by 4 weeks, after the start of pegvisomant but was only significantly higher compared to pretreatment at 24 weeks of therapy (*P* = .01). 25-hyroxy vitamin D levels also tended to increase early after the start of pegvisomant and were significantly higher than pretreatment at 8 (*P* = .05) and 12(*P* = .01) weeks of therapy. OPG levels did not change. Pretreatment serum levels of calcium, 9.35 ± 0.39 mg/dL (mean ± SD), and of phosphate, 3.64 ± 0.33 mg/dL, were normal and did not significantly change with pegvisomant treatment.

**Table 3.** Longitudinal bone turnover marker changes during the first 48 weeks of pegvisomant therapy in 6 patients with acromegaly

	Baseline	4 weeks	8 weeks	12 weeks	24 weeks	36 weeks	48 weeks	*P*-value, baseline vs 48 weeks
IGF-1 (ng/mL)	608 ± 139	362 ± 239	229 ± 144	240 ± 135	204 ± 52	181 ± 25	196 ± 73	.**002**
BSAP (U/L)	32 ± 6	31 ± 5	29 ± 5	28 ± 5	24 ± 5	25 ± 5	22 ± 4	.**002**
OCN (µg/L)	18 ± 5	17 ± 3	13 ± 1	14 ± 3	12 ± 3	12 ± 4	12 ± 5	.**04**
P1NP (µg/L)	69 ± 38	47 ± 16	34 ± 7	32 ± 11	33 ± 15	32 ± 21	29 ± 21	.**004**
NTX (nMBCE/L)*^a^*	17 ± 8	15 ± 9	15 ± 9	16 ± 11	14 ± 11	13 ± 6	15 ± 10	.14
OPG (pmol/L)	5.5 ± 2	4.9 ± 1.7	5.1 ± 1.4	4.5 ± 1.4	5.2 ± 1.9	5.5 ± 1.5	5.0 ± 1.6	.37
TRAP5b (U/L)	1.9 ± 0.5	1.7 ± .39	1.9 ± 0.6	1.9 ± 0.6	1.8 ± 0.5	2.2 ± 0.5	1.7 ± 0.8	.27
PTH (pg/mL)	26 ± 5.5	46 ± 27	35 ± 12	38 ± 16	38 ± 11*^b^*	39 ± 15	32 ± 10	.19
25OHD (ng/mL)	25 ± 8	27 ± 6	36 ± 11*^c^*	32 ± 10*^b^*	33 ± 20	24 ± 9	24 ± 11	.52

Data are mean ± SD unless otherwise indicated.

Abbreviations: 25OHD, 25-hydroxy vitamin D; BSAP, bone-specific alkaline phosphatase; NTX, serum cross-linked N-telopeptides of type 1 collagen; OCN, osteocalcin; OPG, osteoprotegerin; P1NP, intact N-terminal propeptide of type I procollagen; PTH, parathyroid hormone; TRAP5b, tartrate-resistant acid phosphatase 5b.

^*a*^Nanomoles bone collagen equivalents per liter (nMBCE/L).

^*b*^ *P* = .01 vs baseline.

^*c*^ *P* = .05 vs baseline. Significant *P*-values are shown in bold text.

**Figure 2.**
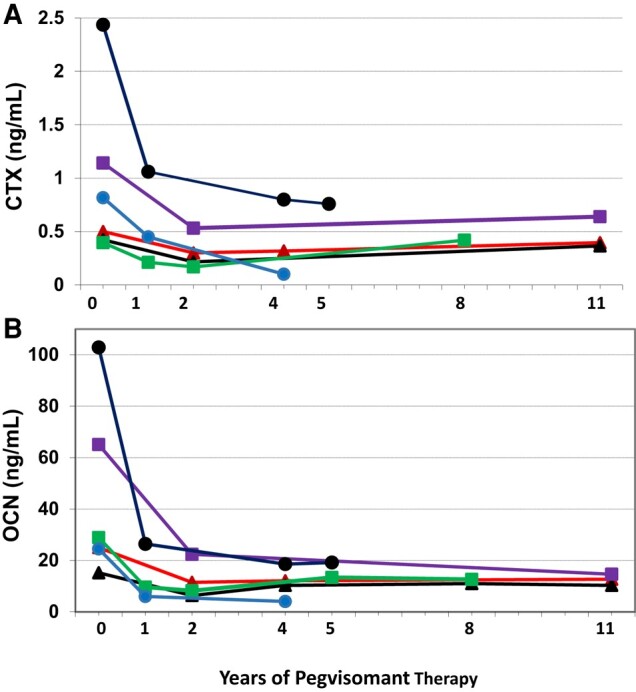
Levels of bone markers, CTX (A) and OCN (B), during long-term pegvisomant treatment in 6 patients with acromegaly. Abbreviations: CTX, C-terminal telopeptides of type 1 collagen; OCN, osteocalcin.

In the additional 6 patients who had levels of CTX, a resorption marker, and N-mid OCN measured before and over time during long-term pegvisomant therapy, levels appeared to remain stable after an initial reduction in the first 1 to 2 years of therapy (Fig. 2).

### Cross-sectional Study

#### HRpQCT

**Figure 3.**
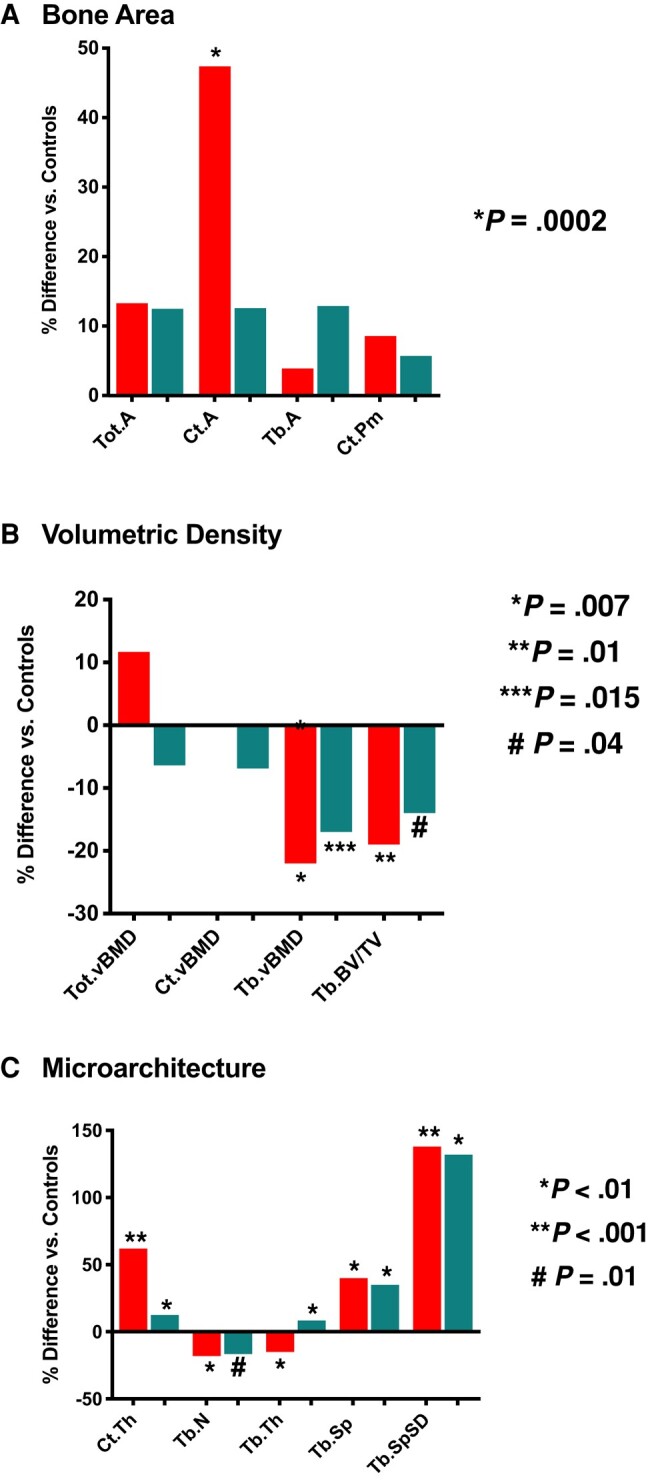
Cross-sectional HRpQCT testing results in 17 patients with pegvisomant-treated acromegaly shown as the percent difference in parameters of (A) bone area, (B) volumetric density, and (C) microarchitecture of the radius (red) and tibia (teal) from nonacromegaly controls. Abbreviations: Ct.A, cortical area; Ct.Pm, cortical perimeter; Ct.Th, cortical thickness; Ct.vBMD, cortical volumetric bone mineral density; HRpQCT, high-resolution peripheral quantitative computed tomography; Tb.A, trabecular area; Tb.BV/TV, trabecular bone volume/total volume; Tb.N, number of trabeculae; Tb.Sp, trabecular separation; Tb.SpSD, trabecular separation heterogeneity; Tb.Th, trabecular thickness; Tb.vBMD, trabecular volumetric bone mineral density; Tot.A, total area; Tot.vBMD, total volumetric bone mineral density.

Acromegaly patients’ HRpQCT parameters compared to those in matched healthy controls are shown in Table 4 (left) and expressed as a percent of those of controls in Fig. 3.

**Table 4.** Results of the cross-sectional HRpQCT study

	PEGV-treated acromegaly	Healthy controls	% diff, PEGV vs CTL	*P*-value, PEGV vs CTL	Remission acromegaly comparison group	*P*-value, PEGV vs Rem.	Active acromegaly comparison group	*P*-value, PEGV vs Act.
Radius								
Total area (mm^2^)	367 ± 105	324 ± 67	13.3	.16	382 ± 113	.69	353 ± 102	.69
Ct.area (mm^2^)	97 ± 28	67 ± 13	47.4	.**0002**	83 ± 21	.10	84 ± 21	.14
Tb.area (mm^2^)	269 ± 114	257 ± 567	3.9	.59	299 ± 109	.44	269 ± 94	.99
Ct.perimeter (mm)	85 ± 11	78 ± 9	8.6	.06	88 ± 15	.53	79 ± 25	.44
Total vBMD (mgHA/cm^3^)	369 ± 123	331 ± 27	11.7	.22	302 ± 59	.05	324 ± 77	.19
Ct.vBMD (mgHA/cm^3^)	853 ± 90	854 ± 25	−0.19	.96	847 ± 74	.81	832 ± 81	.48
Tb.vBMD (mgHA/cm^3^)	137 ± 46	175 ± 25	−22	.**007**	126 ± 47	.47	133 ± 32	.73
Tb.BV/TV (%)	11.8 ± 3.6	14.6 ± 2.1	−19	.**01**	10.5 ± 3.9	.29	11.1 ± 2.7	.47
Ct.thickness (μm)	1.40 ± 0.44	0.87 ± 0.10	62	**<**.**0001**	1.13 ± 0.26	.05	1.18 ± 0.29	.09
Tb.number (1/mm)	1.72 ± 0.39	2.11 ± 0.22	−18	.**001**	1.76 ± 0.55	.83	1.67 ± 0.34	.63
Tb.thickness (μm)	0.079 ± 0.01	0.9 ± 0.01	−15	.**003**	0.06 ± 0.01	**<**.**0001**	0.068 ± 0.01	.**027**
Tb.separation (μm)	0.58 ± 0.19	0.42 ± 0.07	40	.**002**	0.59 ± 0.29	.87	0.57 ± 0.18	.81
Tb.separation SD (μm)	0.41 ± 0.21	0.18 ± 0.07	138	.**0002**	0.26 ± 0.31	.33	0.28 ± 0.23	.11
Tibia								
Total area (mm^2^)	905 ± 218	803 ± 125	12.5	.11	934 ± 204	.69	900 ± 171	.71
Ct.area(mm^2^)	158 ± 33	140 ± 25	12.6	.09	145 ± 65	.14	145 ± 24	.17
Tb.area (mm^2^)	752 ± 214	663 ± 105	12.9	.14	789 ± 204	.99	756 ± 173	.60
Ct.perimeter (mm)	118 ± 14	111 ± 9	5.7	.13	117 ± 19	.44	105 ± 41	.89
Total vBMD (mgHA/cm^3^)	279 ± 69	297 ± 19	−6.4	.31	260 ± 47	.19	257 ± 61	.36
Ct.vBMD (mgHA/cm^3^)	837 ± 72	855 ± 25	−6.9	.31	826 ± 65	.48	802 ± 64	.71
Tb.vBMD (mgHA/cm^3^)	143 ± 47	173 ± 15	−17	.**015**	142 ± 48	.73	139 ± 38	.98
Tb.BV/TV (%)	12.2 ± 3.8	14.2 ± 1.2	−14	.**04**	11.8 ± 3.9	.47	11.6 ± 3.2	.78
Ct.thickness (μm)	1.54 ± 0.30	1.27 ± 0.16	12.5	.**003**	1.39 ± 0.21	.09	1.40 ± 0.28	.11
Tb.number (1/mm)	1.68 ± 0.51	2.02 ± 0.21	−16.6	.**01**	1.82 ± 0.64	.63	1.89 ± 0.54	.50
Tb.thickness (μm)	0.076 ± 0.01	0.071 ± 0.01	8.4	.**007**	0.067 ± 0.01	.**027**	0.063 ± 0.01	.**003**
Tb.separation (μm)	0.59 ± 0.22	0.44 ± 0.06	35	.**008**	0.64 ± 0.01	.81	0.52 ± 0.22	.77
Tb.separation SD (μm)	0.47 ± 0.33	0.20 ± 0.03	132	.**002**	0.43 ± 0.91	.11	0.22 ± 0.18	.86

HRpQCT parameters in acromegaly patients on long-term pegvisomant treatment compared to healthy controls (left) and to 2 groups of acromegaly patients who were matched for clinical features but were in remission or had active disease after surgery alone or surgery followed by long-acting somatostatin analog therapy (n = 4) (right). Clinical features of these groups are shown in Table 2. Data are mean ± SD unless otherwise indicated. Significant *P*-values are shown in bold text.

Abbreviations: Act, active; CTL, healthy controls; Ct.area, cortical area; Ct.perimeter, cortical perimeter; Ct.porosity, cortical porosity; Ct.thickness, cortical thickness; Ct.vBMD, cortical volumetric bone mineral density; F.load, estimated failure load; HRpQCT, high-resolution peripheral quantitative computed tomography; PEGV, pegvisomant; Rem, remission; Tb.area, trabecular area; Tb.BV/TV, trabecular bone volume/total volume; Tb.vBMD, trabecular volumetric bone mineral density; Total vBMD, total volumetric bone mineral density; Tb.number, number of trabeculae; Tb.separation, trabecular separation; Tb.separation SD, trabecular separation heterogeneity; Tb.thickness, trabecular thickness.

Pegvisomant-treated patients had significant differences in bone size, volumetric density, and trabecular microarchitecture compared to controls. At the radius, pegvisomant treated patients had ∼47% greater cortical area (*P* = .0002) (Fig. 3A) and ∼ 20% lower trabecular volumetric BMD (*P* = .007) and trabecular bone volume fraction (*P* = .01) than controls (Fig. 3B). At the radius, trabecular number (*P* = .001) and thickness (*P* = .003) were 18% and 15% lower, respectively, and cortical thickness (*P* < .0001) and trabecular separation (*P* = .002) were 62% and 40% higher in pegvisomant patients than controls, respectively (Fig. 3C). At the tibia, trabecular volumetric BMD (*P* = .015) was 17% and trabecular bone volume fraction (*P* = .04) was 14% lower than controls. At the tibia, cortical thickness was 12% (*P* = .003), trabecular thickness 8.4% (*P* = .007), and trabecular separation 35% (*P* = .008) higher in pegvisomant-treated patients than controls. Trabecular number (*P* = .01) was 16.6% lower in acromegaly than controls. In a post hoc analysis, HRpQCT results were similar when the patients with diabetes were excluded from the analysis. Also in a post hoc analysis, similarly significant trends for differences in most parameters were found when men and women were compared separately to healthy controls (Supplementary Table S1 [39]). Men appeared to be more affected for some parameters, but fewer women were tested in this study. In the multiple regression analysis, the duration of pegvisomant therapy and %ULN IGF-1 were not predictors of HRpQCT parameters.

Pegvisomant-treated patients’ HRpQCT parameters were overall similar to those in the matched remission and active disease groups of acromegaly patients who were not receiving pegvisomant therapy (Table 4, right). Trabecular thickness was higher in the pegvisomant group compared to both the remission (*P* < .001) and active disease (*P* = .027) groups at the radius and to both the remission (*P* = .027) and active disease (*P* = .003) groups at the tibia. Cortical porosity, Stiffness, and F.load at the radius and tibia did not differ in pegvisomant-treated compared to remission and active disease groups of men, women, or overall (Supplementary Table S1 [39]).

Regarding bone markers, levels of OCN and CTX were higher in the active disease than the pegvisomant group but were similar in the remission and pegvisomant-treated groups (Table 2).

Regarding VFs and DXA, of the 17 patients in the cross-sectional study, 5 had fractures. One postmenopausal woman had a grade 1 fracture; 2 men (1 on testosterone replacement and 1 eugonadal) had grade 1 fractures; and 2 men, 1 on testosterone replacement and 1 with unreplaced hypogonadism, had grade 2 fractures. All patients with VF were on pegvisomant monotherapy. In the regression analysis, no HRpQCT parameters or clinical/endocrine features predicted VF. No patients had hip or wrist fractures. One man had hand and ankle fractures related to trauma. DXA results in the cross-sectional study were similar to those obtained at follow-up testing in the longitudinal study and to those in the acromegaly comparison groups (Supplementary Table S2 [39]).

## Discussion

A primary aim of this study was to examine, for the first time, the effects of long-term pegvisomant therapy on bone microstructure of the peripheral skeleton using HRpQCT. We found larger bone size, lower trabecular density, and disrupted microarchitecture at the radius and lower trabecular density and disrupted microarchitecture at the tibia in these patients compared to controls. Since these microstructural abnormalities relate to fractures in other populations, they may underlie the persistent fragility reported in pegvisomant-treated acromegaly [40]. We also found that the microstructural abnormalities in pegvisomant-treated patients are similar to those in acromegaly patients treated with surgery and other acromegaly medical therapies. These data suggest that the microstructural deficits we found are not treatment-type specific. While the pegvisomant and acromegaly comparison groups were matched for a number of important parameters, pegvisomant-treated patients are a subset of acromegaly patients with more difficult to treat disease and longer disease duration since diagnosis. Despite this, we found only higher trabecular thickness at the radius and tibia in pegvisomant-treated patients compared to the acromegaly comparison groups. This suggests that, despite their possible greater exposure of bone to GH/IGF-1 excess, the pegvisomant-treated patients had similarly persistently impaired but no worse bone quality. The fact that pegvisomant-treated patients compared similarly to the remission and active disease comparison groups is consistent with prior studies showing that microstructural abnormalities do not vary with disease activity in surgically treated patients [10, 41]. Although in a recent longitudinal HRpQCT study we found improved but persistent deficits in volumetric density and microarchitecture, Stiffness, and F.load in a group of acromegaly treated successfully with surgery or SRL therapy [10], the current cross-sectional study cannot determine if partial improvements also occurred with initiation of pegvisomant therapy. Overall, although deficits remained in pegvisomant-treated patients, our results suggest that long-term GHR blockade has a similar impact on the bone disease of acromegaly as other forms of acromegaly treatment.

In this study, we also longitudinally assessed the effects of long-term pegvisomant therapy on aBMD as measured by DXA and found improvements at the LS and radius in men but not women. At the hip, however, men had decreases in TH and FN T-scores and aBMD, but none developed new osteoporosis. Our results contrast with those of a prior study that assessed aBMD longitudinally in 5 men and 2 women treated with pegvisomant [17]. Similar to our results, this study found an increase in LS aBMD, but, contrary to ours, it found an increase in hip aBMD by 18 months of therapy [17]. Whether longer pegvisomant treatment contributed to our differing results is unknown. Although the distribution of length of DXA follow-up was similar in men and women, the fact that we studied fewer women may have influenced our findings. Rates of osteopenia and osteoporosis were similar before and after ∼ 7 years of therapy in our study. These data are reassuring, but attribution of clinical significance to aBMD needs to consider that it does not necessarily track with fragility risk in patients with acromegaly [4, 5], including those treated with pegvisomant [18].

Given prior reports of an increased rate of incident VF among treated acromegaly patients, we assessed VF prevalence in the 17 cross-sectionally studied patients. Of them, 2 were found to have a grade 2 VF. Three other patients had grade 1 fractures, but the significance of these is controversial. Additionally, we assessed for VF only by VFA, which has a lower sensitivity for grade 1 than grade 2 and 3 fractures [42]. In a prior study of 35 patients receiving pegvisomant, 33 in combination with SRLs, VF rate was 31.6% and fractures were related to longer duration and active disease and untreated hypogonadism in men [18]. In a prospective study, the addition of pegvisomant to SRL therapy in 35 patients with uncontrolled disease on SRLs decreased incident VFs after a median of 82 months [40]. In a subsequent retrospective study, pegvisomant therapy in 31 patients uncontrolled on SRLs, which were continued in 28 of them, was associated with a decrease in VF after a median follow-up of 38 months [43]. Greater disease activity and prevalent VF were predictors of incident VF [43]. Some of the peripheral microstructural abnormalities we found in pegvisomant-treated patients were associated with VF in 1 [44] but not another [45] study of patients treated with surgery and other acromegaly medications. Possibly due to our study's small size, however, we did not find predictors of VF.

We found, as did others previously, a lowering of BTMs within the first few months of pegvisomant initiation. These prior studies, however, assessed bone marker changes at just 12 weeks [23] or after 7 months of pegvisomant therapy when IGF-1 levels had normalized [16]. We extend these prior studies and provide evidence that BTM levels, after initial lowering, remain stable during long-term pegvisomant therapy. It was clinically important to ensure that long-term GHR antagonism did not oversuppress bone turnover in a GH deficiency-like pattern [19]. In support of this, in vitro pegvisomant did not impair osteoblast function [46]. We also found, cross-sectionally, that BTM levels were comparable in the pegvisomant-treated and acromegaly comparison groups and similar to those reported with other acromegaly therapies [47]. We newly examined the effects of pegvisomant on OPG levels since some prior studies have suggested a relationship of GH-IGF-1 to the receptor activator of nuclear factor kB ligand/OPG system [48-50]. However, in patients with active acromegaly treated with surgery or medical therapy, mostly SRLs, serum levels of receptor activator of nuclear factor kB ligand and OPG levels did not change [51], and we did not find a change in OPG levels with pegvisomant therapy. High bone turnover is considered a key mechanism for the poor bone quality of acromegaly, and, based on BTM data, correction of this persists during long-term pegvisomant therapy.

Limitations of our study should be considered. Interpretation of our results should consider that some of the groups we studied were small in size and patients varied in their length of follow-up. Gonadal function groups could not be examined separately because of the small size of some of these groups. We previously showed that hypogonadism negatively impacts bone microstructure in surgically treated patients, and this is likely to have contributed to the deficits seen in pegvisomant-treated patients. Vitamin D levels were in the 20 to 30 ng/mL range in up to 47% of our patients, which could have been a factor in our findings. However, others found similar microarchitectural changes in vitamin D replete patients [45, 52]. The fact that about one-third of our pegvisomant-treated patients had diabetes mellitus, and our controls did not have diabetes mellitus, could have impacted our findings since a prior HRpQCT study found lower trabecular density at the tibia in acromegaly patients with than without type 2 diabetes [53]. However, microstructural deficits persisted in the patients without diabetes mellitus in our study. While the pegvisomant-treated patients in our study had received other forms of acromegaly therapy and this could have impacted the current state of their bone disease, it is still of importance to characterize bone microstructure in these patients. In addition, most of our patients were on pegvisomant monotherapy, and we did match the comparison groups for concomitant acromegaly medications. In prior studies, 90% to 94% of patients assessed for incident VF on pegvisomant were receiving it in combination with SRLs [40, 43]. Thus, our results are relevant to understanding the bone disease of today's typical pegvisomant-treated patient. We did not measure trabecular bone score, but this would have been of interest.

In conclusion, we have assessed, for the first time, bone microstructure using HRpQCT in patients with acromegaly receiving long-term pegvisomant therapy. We show that these patients have persistently reduced volumetric BMD and microarchitectural abnormalities of the peripheral skeleton. However, the deficits were similar to those found in acromegaly patients not receiving pegvisomant therapy, suggesting that they are not treatment-type specific. They may explain the continued fragility that occurs despite successful biochemical remission of acromegaly. We also found that after an initial lowering, BTMs remain stable and do not appear to become overly suppressed or rise after long-term pegvisomant therapy. Further studies are warranted to understand how to identify, potentially by HRpQCT abnormalities, those patients successfully treated with pegvisomant and other acromegaly therapies who are at continued risk for poor bone quality and VF.

## Clinical Trial Information

Clinical trials no. NCT01809808, NCT03225040.
